# Supplementary material for: Silence of Hippo Pathway Associates with Pro-Tumoral Immunosuppression: Potential Therapeutic Target of Glioblastomas
Source: Cells. 2020 Jul 23;9(8):1761. doi: 10.3390/cells9081761 (PMC7464204; doi:10.3390/cells9081761)
Supplement: Supplementary file 1 [file cells-09-01761-s001.zip › cells-838967-proofreading supplementary/cells-838967-proofreading supplementary figures.pdf]

Supplementary Figures

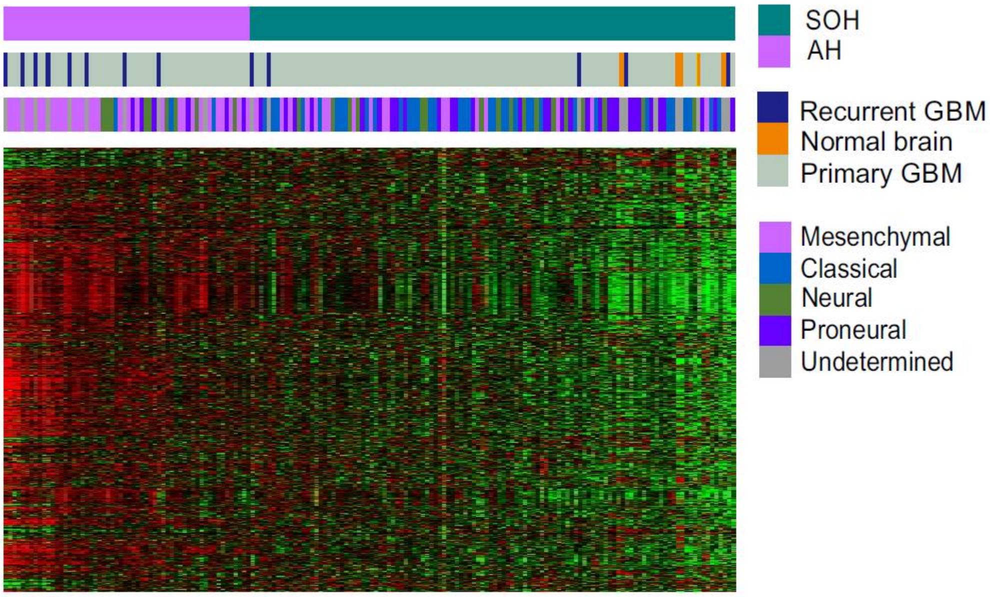

Figure 1. Subgroup prediction in 172 samples of glioblastoma (GBM) from The Cancer Genome Atlas, including 154 primary and 13 recurrent GBMs and five samples of normal brain tissue. As shown in the heatmap, recurrent GBM was more common in the silence of Hippo (SOH) subgroup than in the active Hippo (AH) subgroup, whereas all normal brain tissue samples were classified as AH.

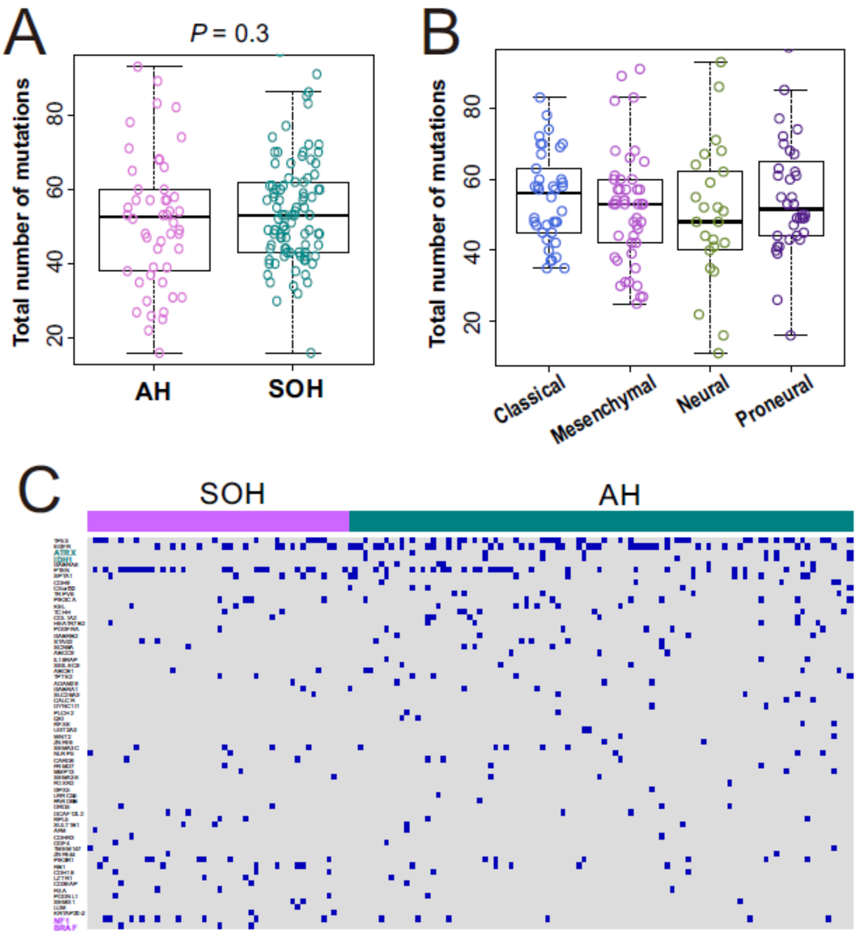

Figure 2. Mutation status in The Cancer Genome Atlas (TCGA) cohort. The 53 genes that are commonly involved in glioblastoma (GBM) were selected on the basis of TCGA study findings. Mutation data were available in 147 samples. (**A**) The total number of mutations, measured as a summation of all mutations in each sample, was compared between the silence of Hippo (SOH) and active Hippo (AH) subgroups, and no statistical difference was found between the two subgroups. (**B**) The number of mutations did not vary much among the four previously recognized molecular subtypes of GBM. (**C**) Mutations in the SOH and AH subgroups are presented in a matrix format in which rows represent individual genes and columns represent individual tumor samples. *IDH1* and *ATRX* mutations were more commonly found in the AH subgroup because these mutations are closely associated in the proneural subtype. *BRAF* and *NF1* mutations were more common in the SOH subgroup.

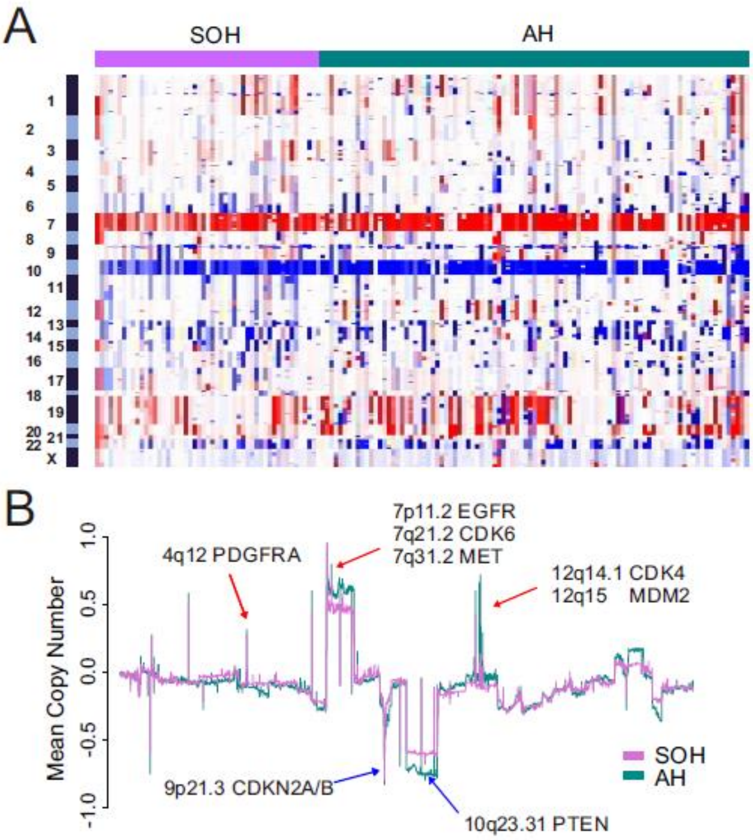

Figure 3. Copy number variation in glioblastoma (GBM) in the silence of Hippo (SOH) and active Hippo (AH) subgroups. Copy number data were available in 148 samples of the 154 retrieved from The Cancer Genome Atlas database. (**A**) The most common amplification was on chromosome 7 and the most frequent deletion was observed on chromosome 10; however, a difference between the SOH and AH subgroups was not apparent. (**B**) Frequent copy number changes in GBM were identified without significant inter-subgroup changes. Copy number variation was less prominent in the SOH subgroup.

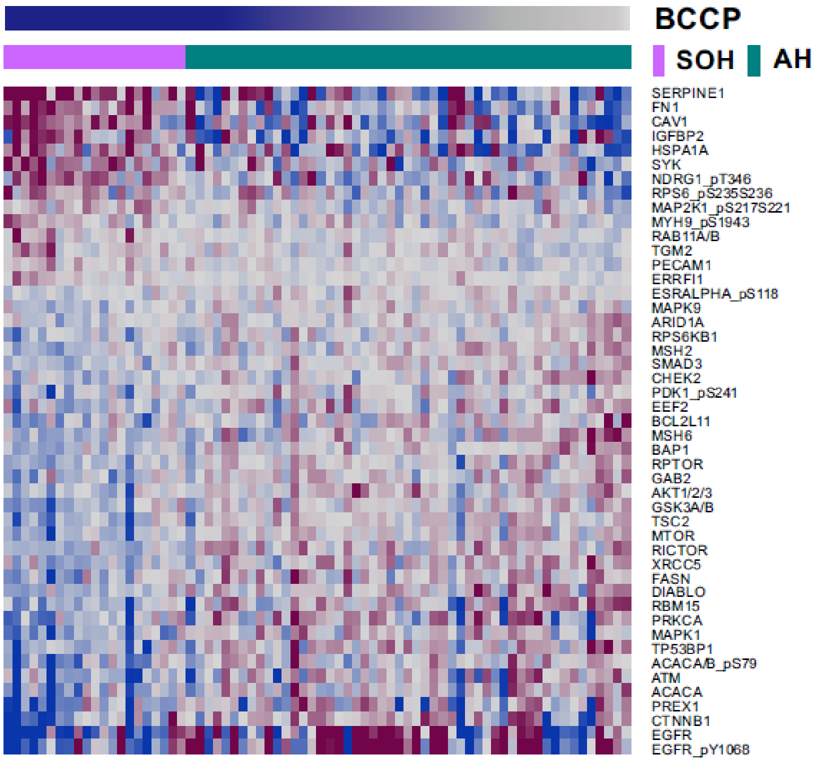

Figure 4. Reverse phase protein array (RPPA) analysis of 72 glioblastoma (GBM) samples from The Cancer Genome Atlas. RPPA data were obtained directly from Gordon Mill's laboratory at The University of Texas MD Anderson Cancer Center. The data are presented in matrix format, in which rows represent individual proteins annotated with gene names and columns represent individual GBM samples. A total of 48 proteins showed differences in protein expression levels between 21 samples in the silence of Hippo (SOH) subgroup and 51 samples in the active Hippo (AH) subgroup. BCCP, Bayesian covariate compound predictor.

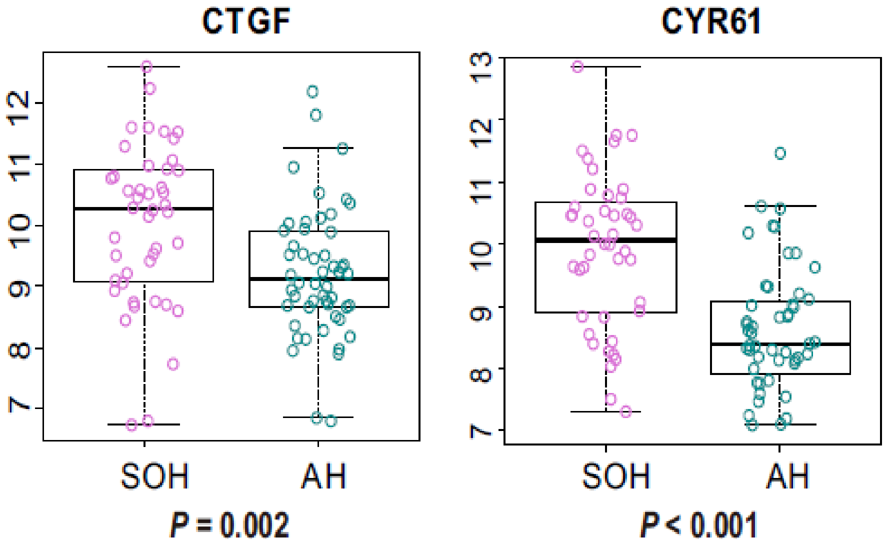

Figure 5. Gene expression levels of downstream targets of YAP1 in GSE16011. *CTGF* and *CYR61* were upregulated in the silence of Hippo (SOH) subgroup compared with the active Hippo (AH) subgroup.

A

Nuclear YAP1 straining	PD-1 Positive	PD-1 Negative
Yes	2	1
No	0	4

P = 0.053 by χ^2 test

B

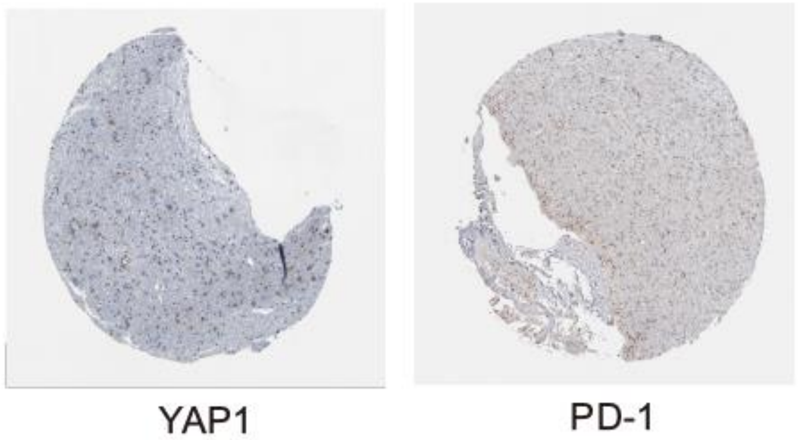

Figure 6. Association of YAP1 activity with increased inhibitory immune checkpoint activity in glioblastoma (GBM). (**A**) Contingency table for nuclear staining of YAP1 and staining of PD-1-positive cells in GBM. Immunostaining data of GBM tissues were obtained from The Human Protein Atlas pathology database (https://www.proteinatlas.org/). Of 2 and 3 tissues stained for PD-1 and YAP1, 2 were stained for both antibodies. (**B**) Exemplary staining image from patient ID 3137. Images were downloaded from the database.

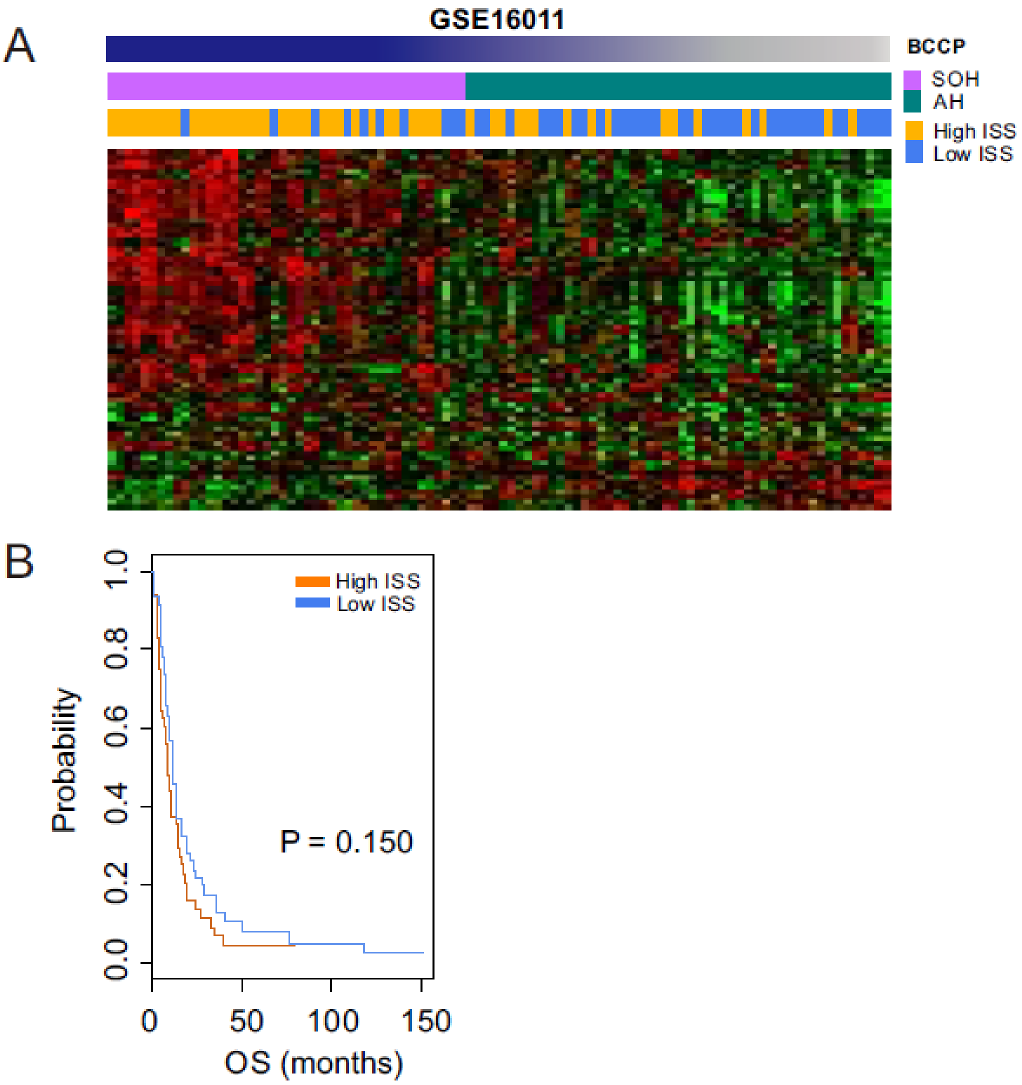

Figure 7. Immune signature of GSE16011 samples. (**A**) In a heatmap showing the immune signature of 96 glioblastoma samples in GSE16011, those with a high immune signature score (ISS) were more common in the silence of Hippo (SOH) subgroup than in the active Hippo (AH) subgroup. BCCP, Bayesian covariate compound predictor. (**B**) Kaplan-Meier analysis revealed that patients with high ISS had poorer overall survival (OS) than those with low ISS, although the difference was not statistically significant.

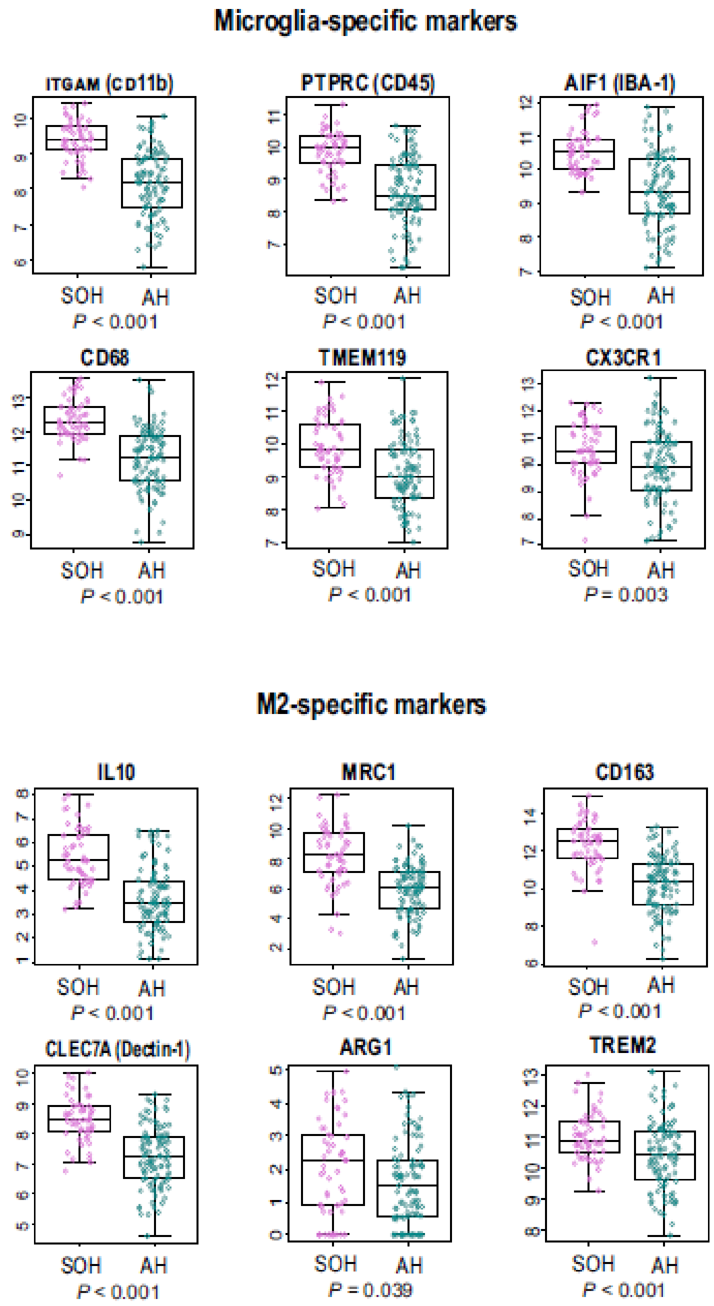

Figure 8. Comparison of gene expression levels of microglia markers between the silence of Hippo (SOH) and active Hippo (AH) subgroups in The Cancer Genome Atlas. Gene expression of most microglial and M2-specific markers was upregulated in the SOH subgroup.

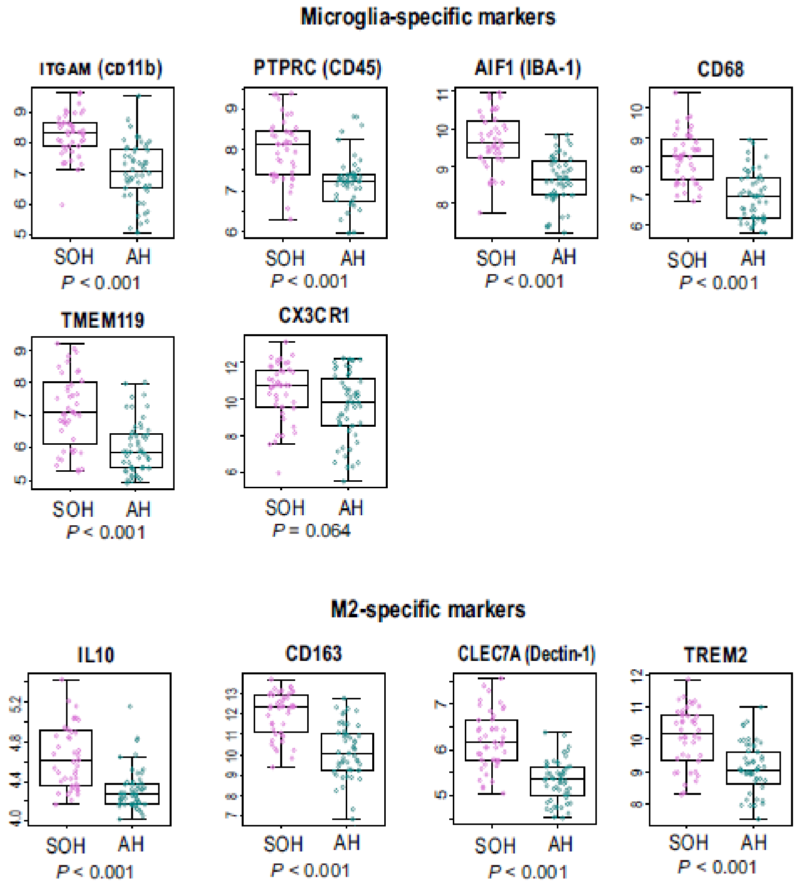

Figure 9. Comparison of gene expression levels of microglia markers between the silence of Hippo (SOH) and active Hippo (AH) subgroups in the GSE16011 cohort. Gene expression of most microglial and M2-specific markers was upregulated in the SOH subgroup.

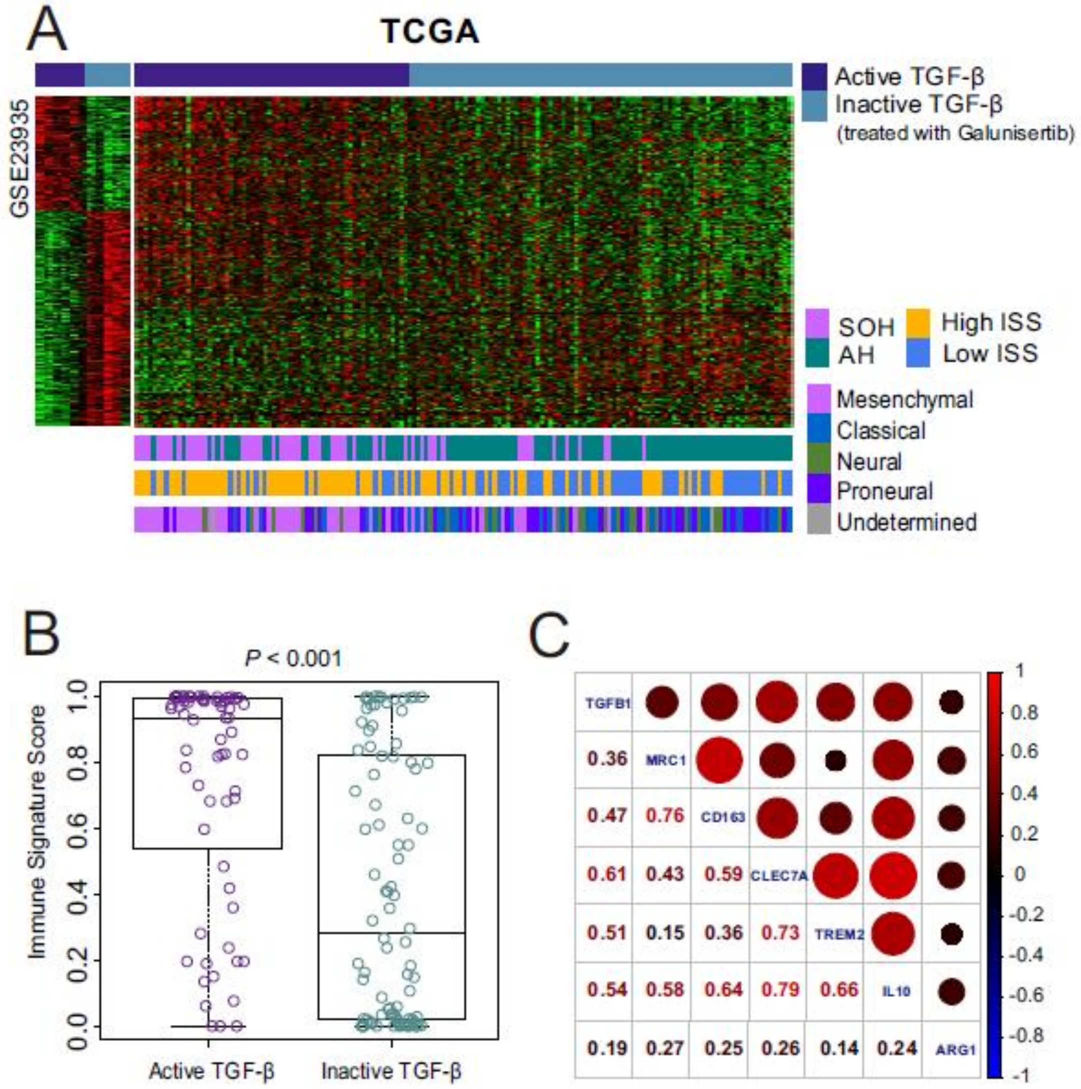

Figure 10. Prediction of the transforming growth factor-β (TGF-β) signature in 154 glioblastoma (GBM) samples from The Cancer Genome Atlas (TCGA). (**A**) The TGF-β signature was predicted in the 154 samples using a 284-gene classifier obtained from GSE23935. These 284 genes were differentially expressed between 11 GBM tumorspheres treated with galunisertib (a TGF-β receptor 1 kinase inhibitor) and 11 controls. GBM samples with the silence of Hippo (SOH) signature were much more common among samples with an active TGF-β signature compared with GBM samples with the active Hippo (AH) signature. (**B**) GBM samples with the active TGF-β signature apparently showed much higher immune signature scores (ISSs). (**C**) The gene expression level of TGF-β was positively correlated with all M2 markers. CLEC7A was the marker most well correlated with TGF-β.

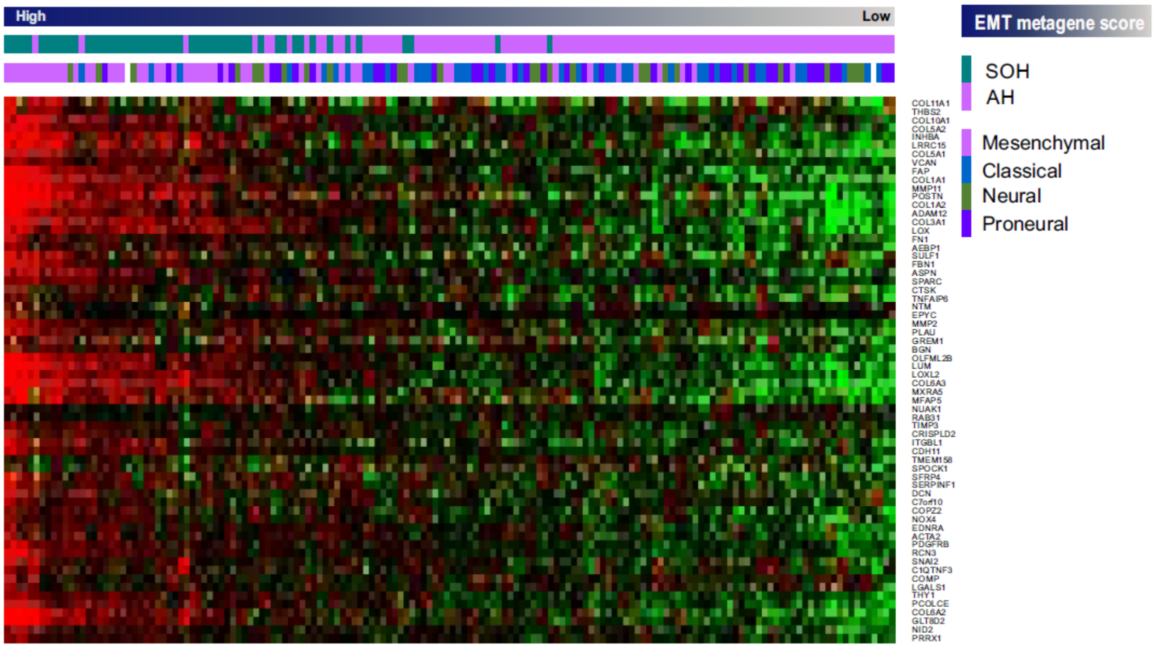

Figure 11. Sixty-four genes of epithelial mesenchymal transition (EMT) signature of TCGA glioblastoma (GBM) cohort. Samples were arranged according to EMT metagene score. GBM samples with silence of Hippo (SOH) and mesenchymal features demonstrate higher EMT metagene score.
