# Supplementary material for: Silence of Hippo Pathway Associates with Pro-Tumoral Immunosuppression: Potential Therapeutic Target of Glioblastomas
Source: Cells. 2020 Jul 23;9(8):1761. doi: 10.3390/cells9081761 (PMC7464204; doi:10.3390/cells9081761)
Supplement: Supplementary file 1 [file cells-09-01761-s001.zip › cells-838967-proofreading supplementary/Table S1.pdf]

Supplementary Table S1. Differentially expressed genes between the silence of Hippo and active Hippo subgroups of glioblastoma in The Cancer Genome Atlas.

Gene symbols	P.value	Fold change (SOH/AH)
PI3	4.04138E-11	10.67630609
MARCO	1.55317E-13	9.818456628
F13A1	9.09925E-18	9.383157381
SAA2	4.63978E-10	9.039005128
CXCL6	3.35466E-16	9.037034675
COL6A3	7.88994E-18	8.58683434
SAA1	1.08407E-08	8.23489957
IL2RA	1.01136E-15	7.925422559
CR1	1.8162E-17	6.842871692
IL8	2.76331E-11	6.718190161
LTF	4.96795E-07	6.697038222
CXCL5	4.50506E-13	6.690275136
PLA2G2A	1.5671E-07	6.205143004
C13orf33	1.51311E-15	6.186861123
COL1A1	1.36394E-17	6.16203136
CHI3L1	1.5153E-10	5.696321453
LYVE1	5.52377E-13	5.567620985
AQP9	8.58241E-15	5.548800915
IBSP	6.051E-10	5.51836307
IL21R	1.07147E-17	5.424683578
IL6	9.79924E-14	5.42012427
THBS1	4.27854E-18	5.372004585
DKK1	2.40917E-08	5.356868302
MMP7	3.09228E-08	5.350809144
CCL18	1.6016E-08	5.350764454
COL13A1	5.14726E-13	5.347621813
MMP1	6.86018E-11	5.294508522
EREG	3.30279E-13	5.140598527
FPR2	4.53998E-18	5.136646056
CCL20	1.64896E-11	5.095704928
MME	1.0991E-12	5.087137144
CCL7	7.93123E-14	5.014669607
GJB2	5.48519E-12	4.98831706
MRC1	2.21007E-12	4.983756523
CYP1B1	1.34539E-17	4.940665808
C19orf59	1.45074E-14	4.911142891
LRRC15	3.13886E-11	4.866465327
EMR1	2.25057E-17	4.823150526
S100A8	3.00053E-14	4.784831873
COL3A1	1.37406E-14	4.734814245
APCDD1L	6.94823E-08	4.639179539
POSTN	2.57658E-05	4.563619198
S100A9	3.28183E-14	4.542302686

CSF3	8.45016E-09	4.527612548
LUM	1.79007E-14	4.42820051
CXCL1	4.54545E-14	4.419031019
HAMP	2.59474E-12	4.416821954
IL7R	2.0894E-15	4.414722893
ALDH1A3	4.33845E-07	4.41180983
HP	4.4802E-07	4.395207001
C7	6.07166E-09	4.298060202
FCGR2B	7.63071E-12	4.297699714
COL5A1	5.86941E-14	4.254037078
CD163	1.40418E-14	4.243319313
BDKRB2	1.10872E-14	4.242369161
HK3	4.94983E-20	4.235035166
SLPI	3.77812E-08	4.215780211
COL6A2	3.88724E-13	4.211197354
AREG	1.60652E-08	4.127027187
TREM1	5.267E-11	4.109246603
SFRP2	1.08969E-07	4.092235507
MCTP2	3.19786E-11	4.069087557
COL15A1	1.23792E-13	4.067661024
COL8A1	3.84825E-09	4.042749495
VDR	2.42485E-18	3.976206456
CCL2	5.31572E-12	3.967804849
COL1A2	4.60271E-14	3.964558667
MMP13	2.11335E-06	3.957383244
ABCC3	7.70522E-11	3.953989786
GALNT5	2.43683E-08	3.944098474
FCN1	1.10605E-11	3.913796278
TDO2	2.148E-09	3.894091171
ADAMTS2	1.07994E-13	3.886784436
IL1R1	4.93401E-19	3.874418499
RARRES1	3.98117E-11	3.866333045
DPT	2.55005E-09	3.859434649
CXCL14	9.10109E-08	3.859368033
TFPI2	1.75695E-07	3.855278702
CLEC10A	2.22663E-10	3.849713253
ACPP	3.24694E-16	3.837495893
FAM20A	2.28454E-19	3.750702135
CXCL3	1.18566E-07	3.728958121
DSG2	1.68542E-05	3.71899078
MYO1G	9.84539E-21	3.68612028
CXCL13	1.23501E-05	3.671039534
CCR2	1.14439E-12	3.658211916
RNASE2	1.58839E-14	3.618554632
CHI3L2	8.09717E-09	3.618225087
MMP9	4.59356E-07	3.615007888
LIF	1.47913E-09	3.61063393

OSM	8.02174E-13	3.584219369
PAPPA	9.46338E-12	3.579821498
SOCS3	5.47101E-15	3.571790445
CD70	1.21962E-07	3.568307487
PODNL1	1.11598E-11	3.558931055
FCAR	1.07922E-13	3.552458794
IL10	2.34198E-13	3.548219739
TNFSF12-TNFSF13	2.28552E-11	3.537297413
PLA2R1	1.90689E-10	3.530895813
EGFL6	1.13894E-10	3.528753533
ADAM6	3.587E-05	3.517030135
STEAP4	2.79687E-12	3.509549515
UBXN10	1.26146E-06	3.486189652
ADAMTS14	2.8771E-12	3.470167173
CFB	1.50315E-09	3.468142886
THBD	1.96201E-22	3.433003425
ANPEP	1.65236E-18	3.420874103
LILRA5	7.40474E-11	3.420699869
STC1	7.78715E-12	3.40156354
TFAP2C	4.61668E-09	3.395671062
ANGPTL4	3.11497E-09	3.384777944
FCGR2C	7.3326E-16	3.36575155
PTGS2	2.87015E-10	3.360774102
JAK3	1.98267E-18	3.350696796
NNMT	1.63739E-07	3.343880381
ASGR2	4.86699E-18	3.34222566
MLPH	7.16959E-09	3.330529197
MXRA5	1.11268E-10	3.328078071
PTX3	2.81743E-09	3.323612667
FOLR2	1.85349E-11	3.311486023
FBLN2	3.2947E-09	3.302634694
BCL2A1	1.53212E-12	3.292793459
LILRB2	1.57189E-17	3.286068115
CCL13	3.84484E-09	3.282281814
KRT17	1.18135E-07	3.26243532
LOX	2.90065E-10	3.260417038
FPR1	3.81152E-13	3.256616815
MYBPH	1.00551E-05	3.251657307
BIRC3	1.03682E-09	3.248709425
LTBP2	5.79099E-22	3.245869667
CTHRC1	5.81525E-09	3.245738578
ADAM8	4.16948E-12	3.241824261
TMEM150B	1.42377E-12	3.236310914
COL10A1	1.85838E-09	3.228761904
VENTX	5.80341E-15	3.224732642
DSC2	1.14497E-14	3.221547417
PDZK1IP1	7.52308E-08	3.216336713

SERPINE1	4.18129E-10	3.2148329
SERPINA1	1.82998E-15	3.205940802
RNASE3	5.68834E-12	3.205651698
MET	2.26358E-06	3.199009518
ESM1	1.27136E-07	3.193385116
FAM176A	1.81479E-07	3.188575433
PTGES	6.9436E-11	3.187596601
AMICA1	3.71106E-12	3.179424798
TIMP1	1.77217E-14	3.172535293
CD209	2.39985E-07	3.169632832
COL12A1	5.3142E-09	3.165786624
MSLN	9.27844E-06	3.165478617
PRF1	1.10695E-12	3.158255525
HRH2	3.97235E-14	3.158117322
CHRDL2	1.30536E-05	3.155891506
IGFBP6	1.76235E-11	3.142764008
LILRA6	1.4224E-12	3.14136106
P4HA3	6.65609E-13	3.139379691
KCNJ15	2.00939E-14	3.132034922
ISLR	1.0653E-10	3.120553069
WNT2	1.0993E-07	3.112963942
MFAP5	5.06677E-06	3.09369282
IL1R2	1.4003E-06	3.082775667
FAM83G	1.81602E-10	3.081352894
LRRC8E	1.94727E-08	3.080830132
ABCA13	6.50587E-06	3.080252038
HSD11B1	1.8747E-09	3.073705109
AIM1	3.63359E-11	3.071805672
FAP	8.58287E-09	3.071034292
CHIT1	3.18742E-06	3.064038866
IL1B	4.75565E-11	3.061212259
C15orf48	3.82236E-09	3.059442557
IER3	2.26782E-20	3.058165941
TREML2	1.34338E-16	3.054958919
CP	7.77007E-08	3.054213422
CD300E	2.24852E-17	3.048282043
LILRA3	7.14383E-15	3.039167228
IL1RN	6.54521E-11	3.038710447
C5AR1	1.82702E-16	3.03377202
TLR8	3.95179E-12	3.031519276
POPDC3	8.78889E-08	3.030713666
SRPX2	1.18394E-10	3.030270928
IGJ	0.000273749	3.02514473
SCNN1B	0.000138045	3.023270701
CEACAM4	5.1696E-11	3.019578363
HAS1	4.11968E-12	3.006649776
MS4A4A	4.94316E-15	3.004665366

ROR2	1.93024E-08	3.003382964
C1S	2.26136E-12	3.001328326
LRG1	2.73415E-15	2.994702353
NFKBIZ	7.3371E-14	2.992459749
ICAM1	7.79289E-15	2.991669401
GPR84	6.21528E-14	2.982763667
CD14	5.84286E-17	2.948956439
ARHGEF5	3.65816E-12	2.947333131
HSPA7	8.19508E-12	2.947279491
SPOCD1	5.51539E-07	2.947129837
S100A12	3.59017E-09	2.946834823
SLAMF8	5.77475E-15	2.943867599
EMB	4.45833E-18	2.939405444
LILRB3	6.10846E-14	2.937795953
CDCP1	1.133E-12	2.933050318
PTGFR	1.1477E-06	2.931942137
SCIN	9.04161E-11	2.929533563
ALPK2	1.24998E-05	2.926398986
TNFRSF8	8.10188E-14	2.926143275
RFX8	3.98241E-10	2.920431948
PCOLCE	3.84341E-10	2.9198034
MAP3K8	6.43297E-20	2.911861418
PTPRN	2.57906E-05	2.905704038
OGN	2.02885E-05	2.901287802
AHNAK2	2.63843E-07	2.901008947
ADAMTS12	2.97164E-08	2.899431818
BTBD11	2.03832E-07	2.891762906
TNFSF14	1.12779E-09	2.887163074
GCNT1	5.51751E-12	2.886947986
CXCL2	4.33473E-08	2.885639002
FCGBP	1.32289E-10	2.881094965
KLRB1	3.28705E-08	2.874727692
STAB1	6.09424E-21	2.872889667
STRA6	3.95388E-09	2.866579149
PCSK1	1.23515E-07	2.861313904
FCGR3B	1.15123E-07	2.841934538
TGFBI	4.79042E-13	2.836869939
C5orf46	2.97153E-08	2.83412445
FPR3	4.27142E-12	2.826566236
DOCK5	4.57074E-10	2.826171545
CLDN23	8.60564E-12	2.822601211
LYZ	1.0084E-09	2.815851531
BATF	3.05946E-14	2.814815022
SLC11A1	1.6341E-14	2.811498591
GNA15	1.40002E-18	2.808068527
GZMA	1.58558E-07	2.801340795
CPA4	1.0592E-07	2.800848022

INHBA	4.21567E-09	2.796601073
PLAUR	8.19553E-20	2.788730948
IL18R1	1.82799E-11	2.788432618
CD3D	6.15267E-07	2.782567621
CSTA	2.68712E-11	2.77245359
GPR77	3.80524E-13	2.77131334
CPZ	9.77796E-06	2.771242809
GZMK	5.61416E-07	2.768185677
VSIG4	1.97283E-11	2.767533777
PTGER2	1.07009E-12	2.76621645
C5orf49	0.000125086	2.757753
CA9	0.000643124	2.754286754
LILRB5	1.82658E-06	2.751550873
SIRPB2	1.76943E-13	2.749263487
EMR2	6.86014E-19	2.744840493
CH25H	2.26387E-09	2.744483494
INSRR	6.24365E-07	2.739087472
ITK	5.65032E-09	2.737386682
CXCR1	8.17942E-07	2.731729949
CES1	0.000346208	2.731458797
CFD	9.54233E-10	2.728929811
OLFML2B	2.1074E-15	2.72875121
NLRP12	9.59792E-14	2.728119759
DOK2	2.93618E-12	2.723600959
RNASE1	1.05345E-10	2.723415742
IL31RA	4.28586E-08	2.722669058
CSF2RB	4.5001E-18	2.718704557
IL2RB	4.75478E-13	2.717600648
SH2D2A	3.08581E-14	2.716425606
SELE	6.69792E-09	2.707431271
DPP4	6.0153E-06	2.705408959
TACSTD2	2.15911E-09	2.705366764
SLAMF6	1.20672E-10	2.70489427
FCGR3A	9.02383E-15	2.701271695
ST14	4.07388E-13	2.700046637
TPBG	1.1639E-06	2.695645804
CCL26	3.16693E-10	2.691749268
CXCR6	2.0803E-08	2.690874293
SERPINB2	1.14382E-07	2.690577791
LBP	0.000236095	2.68978231
ITGA8	4.5253E-07	2.686990968
RHOD	5.28705E-05	2.685487853
FNDC1	0.000157362	2.68509579
UBD	1.8305E-06	2.680716948
FAM23A	3.55825E-10	2.679164662
CCL8	9.75705E-08	2.678645341
NOD2	3.10382E-15	2.677171351

PLAU	6.12811E-11	2.676819721
CATSPER1	7.67067E-09	2.665487353
KCNN4	3.86867E-07	2.654662079
ACAP1	8.42818E-12	2.652874447
BDKRB1	6.90446E-11	2.652361114
CFI	1.15066E-10	2.647000543
CD2	1.63788E-07	2.645497018
TSPAN2	5.69986E-07	2.640914314
KRT18	3.30576E-09	2.631792831
CCL3	7.05049E-06	2.629418702
KRT80	2.14722E-07	2.629264147
LOXL1	6.56223E-09	2.628716136
C1R	9.08241E-12	2.62372082
SAA4	1.17565E-06	2.623156268
TMEM26	2.9057E-12	2.623081151
FHL2	1.41865E-09	2.621111731
LAIR1	3.374E-17	2.620179425
ALOX5	1.91957E-15	2.61825568
SNX10	9.22996E-08	2.618084712
HLA-DRB6	5.71623E-05	2.614082698
FBLIM1	1.08114E-10	2.610151457
MT1H	2.84548E-06	2.607804854
FCGR2A	5.5626E-18	2.60703896
SPP1	8.9019E-08	2.606200311
TWIST2	4.6245E-08	2.601377565
OR51E1	2.3353E-06	2.600908633
ALOX15B	0.000185655	2.5998708
C6orf141	0.000177097	2.598555753
ACTG2	1.05851E-06	2.594175072
SLC16A10	4.97145E-08	2.590026015
GPR183	9.50502E-12	2.589570614
LRRC25	5.5072E-17	2.58742564
CEACAM21	4.39586E-12	2.585645902
MMP8	7.56954E-07	2.584884508
VGLL3	2.50119E-10	2.583174468
KYNU	1.06869E-14	2.582694053
CYGB	1.35299E-09	2.582106298
SIGLEC9	1.25639E-15	2.581182485
MSC	1.54307E-08	2.576537731
IL10RA	4.29066E-16	2.575676843
LCK	1.71383E-08	2.574553682
DIO3	3.45417E-07	2.573656149
CAMP	3.41533E-05	2.572320603
LOC100233209	1.53147E-12	2.571476857
RUNX2	1.26411E-14	2.571413127
ASPN	4.03111E-08	2.569485609
NIPAL2	7.57272E-12	2.566592206

DKK2	1.39928E-07	2.566519391
SCNN1G	0.00016618	2.564125206
PTPN22	4.12495E-12	2.563883173
CD200R1	6.69437E-11	2.562506228
TEC	1.03982E-14	2.561282811
MS4A6A	2.24332E-12	2.556656518
C20orf200	1.00971E-05	2.556588256
GPR171	2.32268E-12	2.556299252
GATA6	5.2011E-06	2.554704848
CMKLR1	3.38122E-16	2.552424172
CTSC	3.41245E-18	2.551342436
PPBP	8.5341E-06	2.549473325
MAN1A1	5.44193E-13	2.549194055
CLEC5A	3.40704E-07	2.548869185
RGS18	2.22132E-10	2.547432318
AIRE	9.59703E-08	2.545982712
TNFAIP2	2.57125E-10	2.541391936
VNN2	4.37449E-10	2.540688052
SIGLEC7	6.63156E-12	2.53931051
MAFB	2.00709E-15	2.539128094
CD3E	1.03294E-07	2.537741178
ITIH2	7.82781E-05	2.536889196
LOXL2	6.82145E-12	2.536011213
CTSZ	2.05522E-18	2.5328314
SIGLEC10	1.94376E-11	2.532589255
NDRG1	3.70553E-10	2.528581867
CD96	2.76407E-09	2.527720113
G0S2	1.22575E-06	2.526897721
TNFRSF1B	9.92642E-20	2.52398266
TNFRSF18	1.48856E-08	2.520696016
CCR5	9.61165E-12	2.518200496
HOXB6	9.97761E-05	2.517269461
PCOLCE2	9.13585E-06	2.515836905
IGFBP3	2.28821E-06	2.515777355
FER1L4	8.32737E-05	2.511280293
CCR7	6.90499E-12	2.511278439
SIGLEC14	7.83246E-07	2.510180516
SLC16A3	9.7962E-15	2.510087183
FEZF1	0.00066307	2.509897489
SULF1	0.000318306	2.509865704
CLEC12A	4.48556E-06	2.509051347
PTGER4	1.40703E-14	2.505678258
MGP	3.64692E-07	2.504480837
C9orf44	0.000141006	2.502426029
ITGAM	3.50229E-14	2.501357482
MAPK13	1.55318E-12	2.500343185
KMO	7.59187E-09	2.497042476

SLA	3.38131E-14	2.493287652
OBFC2A	4.1705E-16	2.492160241
RAB11FIP1	1.32097E-15	2.48967163
IL1A	1.16023E-08	2.487572135
GAL	0.000520745	2.487564404
RCAN2	5.15786E-07	2.487236712
CYR61	3.09512E-09	2.485239673
FABP4	7.31334E-05	2.484016284
PRG4	6.53377E-05	2.472454538
LILRB1	2.34361E-12	2.468512912
FOSB	0.000171989	2.46581158
HTR7	5.31586E-14	2.465725819
TMEM61	1.09461E-08	2.463422469
EMR4P	4.37613E-08	2.462197195
LY96	1.92756E-10	2.462046236
TNFRSF11A	8.52403E-12	2.460668016
COL14A1	8.64322E-05	2.460149348
COL24A1	1.81103E-05	2.460126841
SRGN	1.31162E-12	2.459477217
IFI30	2.37698E-13	2.455522511
CLCF1	1.00821E-08	2.454546108
KRT7	7.20014E-06	2.454327178
SLAMF9	0.000125313	2.453092562
MMP11	1.60911E-09	2.450481259
PLIN2	1.71916E-11	2.449029042
CD163L1	1.83852E-07	2.446518431
STAT4	2.20605E-07	2.446101518
KIAA1199	3.95939E-08	2.442553061
C2	2.84773E-10	2.437064293
HLA-DQA2	0.000343841	2.434346593
SLC17A9	1.44085E-14	2.434143696
CLEC2B	3.09623E-09	2.431792511
GPR141	2.44609E-10	2.43035834
CD93	3.06907E-17	2.430212591
S100A4	3.48582E-09	2.428112474
SUSD2	9.7781E-15	2.425870301
C3	8.47528E-10	2.42246595
SLAMF1	3.22208E-11	2.422252092
ADAM12	5.83799E-09	2.422203932
GPR109A	6.70875E-08	2.421160784
CHRNA9	0.000262407	2.419490804
CTSK	1.1175E-08	2.419195276
TSLP	7.98672E-06	2.417998418
C1orf38	3.72E-15	2.417784345
CLEC7A	2.95665E-13	2.415963071
IL4R	3.1752E-27	2.415262449
CD300LB	9.73338E-15	2.414920656

TIMD4	1.80427E-07	2.412803168
ITGBL1	1.07682E-06	2.409666571
TREML3	3.54811E-07	2.409129735
SCN9A	0.000710937	2.400109495
AOAH	3.42312E-09	2.398385369
ALOX5AP	1.01734E-09	2.398255853
GPR132	1.3223E-17	2.397723795
MS4A7	1.14576E-12	2.396079754
LAMB1	6.86559E-11	2.393012678
PTPRC	3.81793E-12	2.390551088
LOC100272216	1.51212E-08	2.388364985
CLDN11	4.38209E-05	2.387901506
PTPRU	7.98279E-07	2.387158342
SVIL	1.31928E-11	2.385384725
SIGLEC12	7.28262E-06	2.385278458
SLC2A5	8.9114E-11	2.384470498
LCN2	2.56625E-06	2.384225572
CYTIP	4.19749E-12	2.381492985
OR2A7	4.63418E-10	2.376764163
COLEC12	6.46534E-08	2.3753839
CCL14	1.6566E-05	2.374748903
MNDA	1.05736E-12	2.374086363
TAGLN	3.71204E-09	2.373228877
PI15	7.97195E-07	2.371684825
WISP1	0.000255209	2.369934948
CD52	1.9775E-07	2.369639597
SLIT3	6.26535E-06	2.368018278
CXCL12	5.67285E-09	2.367555666
ASAM	2.93377E-05	2.367535126
C1QA	9.88241E-12	2.364370182
CD5	5.07197E-09	2.363757305
BCL3	5.37681E-14	2.361125955
CTSW	7.84622E-08	2.359448104
RHOH	8.16418E-13	2.358464643
SOD2	7.38799E-08	2.357459385
CD244	7.12758E-10	2.357061831
RETN	4.70017E-07	2.355983586
C3orf52	1.34527E-08	2.355279863
IL11	1.04579E-05	2.354074389
IL15	5.39638E-09	2.351197746
CD33	8.37602E-14	2.35116351
DPYD	4.2683E-10	2.350876846
ME1	2.96875E-06	2.350773318
GZMB	1.11427E-06	2.350439039
CACNA2D4	4.36789E-14	2.350346259
HCK	4.9146E-14	2.349368981
FN1	6.1896E-16	2.349084041

FCER1A	0.000266936	2.348691335
CLEC4D	2.17914E-10	2.348430546
SEMA3C	1.75967E-05	2.345585644
CLEC17A	0.000145604	2.345507603
CST7	9.40972E-10	2.345436444
TGM2	4.13405E-16	2.344514911
PSTPIP1	4.1477E-11	2.343784765
HMOX1	4.39745E-11	2.341669734
TNFRSF10D	5.07678E-14	2.341659955
MMP3	0.000102703	2.34160001
AQP3	2.43693E-10	2.341545176
SNAI2	1.63461E-09	2.341332975
OSCAR	6.83086E-13	2.340752212
CNTNAP3	2.7726E-07	2.33952138
GLIPR1	7.08144E-13	2.338132151
FYB	1.162E-10	2.33784597
ITGB2	1.07906E-13	2.334260557
CXCR3	2.26343E-07	2.333282716
CSF3R	8.33291E-14	2.332194331
CRTAM	3.05154E-09	2.330479597
AIF1	1.41962E-11	2.330168525
PHLDA2	3.64413E-06	2.328923912
GIPC2	1.51566E-10	2.328091526
FCGR1C	9.9311E-10	2.325816532
CCL3L1	0.000910857	2.324855625
MSR1	4.57403E-11	2.322747675
ABCA6	0.000128838	2.318173683
DARC	0.000322235	2.317116319
RGS4	4.31924E-05	2.313171917
SNAI1	2.45854E-07	2.311847068
CYP11A1	0.00010097	2.310601322
CNN1	4.6979E-07	2.310364985
FMNL1	4.54577E-14	2.3102658
CTSS	2.46937E-12	2.307773573
CPXM2	0.000761786	2.305678425
KRT8	3.32777E-07	2.305233738
HSPA6	1.42016E-08	2.303721664
TNFRSF10A	8.51981E-11	2.303144773
C1QB	3.38378E-11	2.302118006
CCL4	1.9279E-05	2.302100225
ARHGAP30	2.2226E-15	2.301199771
GPR109B	1.98991E-07	2.300939861
P2RY8	5.31542E-10	2.298881518
PKP1	2.87321E-05	2.297722032
OMD	8.03008E-05	2.296859471
SLC6A6	5.14897E-11	2.296457818
ENTHD1	1.29265E-06	2.294640194

DUSP1	8.18983E-09	2.290819882
CYTH4	5.93702E-16	2.289301921
MYL9	6.75421E-13	2.288944299
SPHK1	1.58933E-10	2.28769389
CCR1	8.61982E-13	2.286947502
MICAL2	7.7775E-12	2.286792587
CSF1R	9.79424E-13	2.283853559
TYMP	1.10431E-11	2.283673865
TNFAIP3	1.08057E-16	2.282842398
PTPRH	4.53822E-05	2.282469101
CD226	5.03917E-11	2.280233655
ITGA5	2.67068E-17	2.279199542
DOCK2	4.86278E-13	2.279077746
VAV1	1.40347E-12	2.278618096
LCTL	1.45359E-07	2.278022551
MOCOS	2.4695E-05	2.276720252
LRRC2	0.000170432	2.276337418
LAPTM5	5.40409E-13	2.275645536
ADAMTSL1	0.0004535	2.274465431
TLR2	9.48115E-13	2.274168535
CD248	3.0982E-11	2.272950921
C6orf105	6.59873E-08	2.270651555
CD300C	1.16051E-11	2.269947718
IL24	3.40999E-08	2.269240221
CCL5	9.0813E-09	2.268977853
RPSAP52	9.52457E-05	2.267873124
CD300LF	2.43812E-09	2.267693806
CD7	7.63133E-10	2.267658761
PIK3AP1	5.57534E-12	2.266706663
AMPD3	3.49343E-14	2.266562436
POM121L9P	2.82429E-06	2.266437265
C6orf97	9.06469E-06	2.266357045
TCHH	5.84945E-10	2.264035854
TRPM2	4.28582E-16	2.262382775
ORM1	1.25464E-06	2.262358724
ADAMTS4	1.53687E-10	2.262075154
CHSY3	4.9491E-09	2.260939429
FAM83H	6.98974E-10	2.260030391
KCNK6	3.57489E-18	2.259710946
CRISPLD2	3.19732E-12	2.257560946
EPB41L3	1.2827E-09	2.257231825
C2orf40	7.16873E-05	2.25687008
FGL1	3.74318E-06	2.25372754
FCER1G	2.71523E-12	2.253516878
CASS4	6.9013E-11	2.252131461
BNC2	7.87433E-07	2.251418919
PIK3CG	4.11115E-10	2.251399941

TLR5	7.60989E-13	2.250525686
SNX20	7.60795E-12	2.250437753
HLA-DOA	1.91557E-10	2.250298816
ACP5	3.49311E-07	2.249925014
PLB1	2.82903E-13	2.249342414
ADAM28	4.48634E-09	2.247490692
HLA-DRA	5.32953E-09	2.24635512
CD68	2.50775E-14	2.245826633
MFSD7	8.99916E-10	2.243579037
ICAM4	1.47054E-10	2.24319617
EGFLAM	6.45831E-08	2.242060096
C1QC	1.98528E-11	2.241290839
KIAA0748	6.80134E-06	2.241040742
B4GALT1	4.42302E-16	2.239469162
IGF1	1.76686E-08	2.239133372
ICOSLG	1.43509E-12	2.238956124
NCKAP1L	4.67435E-12	2.237674651
FOSL2	2.4284E-13	2.236117795
TSHZ2	2.41111E-05	2.234386626
SYNPO	5.81927E-10	2.233344632
KISS1R	0.000453892	2.231703747
TNFRSF9	6.52035E-09	2.230890148
AFP	0.000120827	2.230190666
TNFSF9	5.95462E-09	2.22953234
P4HA2	1.51005E-12	2.229313432
NRP1	1.74762E-16	2.22906571
VNN1	3.40805E-09	2.227538654
GJA5	2.55702E-10	2.22753815
GXYLT2	3.47821E-10	2.226584646
PRKCDBP	8.55794E-09	2.226495443
OSMR	5.62503E-08	2.225726137
TNFSF8	8.5645E-09	2.224563624
ARHGEF35	9.62086E-09	2.224191044
TNFRSF10C	5.66206E-12	2.223212474
SFN	1.60757E-05	2.222593348
FBP1	8.82627E-10	2.222552178
CXCR2	1.76667E-08	2.222458561
RAC2	1.26764E-14	2.222343826
AHR	8.83202E-08	2.221738133
SKAP1	4.18929E-07	2.221547562
CTSB	7.59212E-20	2.220253471
COL7A1	1.46597E-05	2.219198613
ANKRD1	7.35109E-06	2.219101919
DCN	4.27527E-06	2.21673555
PIK3R5	2.8316E-14	2.215333044
SYK	6.62738E-15	2.21440237
CD1D	1.0477E-09	2.21432759

MPO	1.06703E-05	2.214197219
PTPN7	1.0006E-12	2.211569867
CNN2	4.49564E-09	2.211413643
FAM26F	1.40598E-07	2.211037525
AGPAT9	1.9358E-08	2.210961942
SERPINA5	6.12159E-06	2.210924124
CD22	0.000139849	2.209896763
NAMPT	4.83E-07	2.209208984
COL6A1	8.48418E-09	2.209208712
FCGR1A	2.64347E-09	2.209016584
FAM177B	8.48795E-07	2.206941951
SSC5D	1.68925E-06	2.203493983
MYO1F	2.10656E-15	2.201659864
IL18RAP	4.51002E-07	2.201546886
HEPH	1.94053E-07	2.200627917
CASP5	5.47829E-08	2.199997532
C8orf4	4.79455E-08	2.198759071
PRR16	2.97432E-09	2.198176143
NCF4	3.49826E-13	2.198167832
ARL11	4.54317E-11	2.19731944
PTAFR	3.50279E-08	2.197117464
ATP8B4	4.81058E-11	2.193814961
S100A11	1.40271E-12	2.192820466
C11orf88	0.000904923	2.191694765
SPINT1	5.20426E-10	2.189349031
GPR97	1.5645E-06	2.188803856
RBM47	3.22946E-11	2.188488188
CA12	1.51439E-05	2.188269343
S100P	9.44041E-06	2.187495995
GALNT6	7.00277E-11	2.187298661
LTBR	6.34815E-17	2.187050114
GPR65	8.11793E-10	2.186872529
ADAMTS1	8.52545E-10	2.184793443
MFAP2	5.85119E-05	2.184683934
CD3G	2.54587E-07	2.183546632
WDFY4	1.64316E-08	2.183513495
SGMS2	1.43156E-08	2.179760113
C13orf26	0.000270875	2.178897496
CYBB	7.23852E-10	2.176442229
CELSR1	0.00034115	2.176191239
NCF1C	2.5235E-08	2.175880052
ADAMTSL4	1.23697E-09	2.175518499
ITPR3	8.83906E-06	2.175160907
HBA1	0.000463698	2.174966389
C2orf89	3.12514E-08	2.174810723
SELL	3.81168E-06	2.174779211
RHBDF2	2.48892E-13	2.174014515

ACTA2	3.74274E-10	2.171884994
LY75	1.49626E-07	2.171741163
TWIST1	5.8621E-05	2.171127754
RGS1	1.06663E-05	2.170634644
RASGRP4	1.42376E-12	2.170446954
CD4	2.64744E-15	2.169471002
TNFSF11	8.32081E-12	2.168987537
NFAM1	1.1768E-13	2.168452304
CD55	1.45166E-15	2.165613757
TNFAIP8	9.43221E-14	2.164675044
CYP7B1	9.63504E-06	2.161470774
LOC283050	1.59658E-09	2.161245675
C4BPA	0.000180915	2.160719361
DENND2D	2.0561E-10	2.156534527
BACE2	9.28931E-12	2.156520355
CIITA	8.92352E-09	2.156404109
FCGR1B	1.05635E-08	2.155265092
PLCB2	1.9328E-12	2.155173681
LSP1	2.13475E-11	2.15307254
HCLS1	3.30839E-14	2.152294993
C4BPB	2.18074E-13	2.152001881
EXOC3L2	2.86773E-06	2.15188606
SERPING1	8.06951E-09	2.151607393
CD40	2.41162E-15	2.151447549
FLJ36031	4.38294E-07	2.149689221
ASS1	1.75518E-05	2.149656403
VAMP8	1.54892E-09	2.147154346
ELF5	6.05887E-05	2.146920271
PPL	0.000295817	2.145888098
FAM179A	0.000383618	2.14396442
TNF	5.60462E-06	2.143075897
CLCA2	9.37436E-06	2.142405599
IL2RG	1.41675E-09	2.141571284
LCP1	4.74229E-15	2.140869923
SASH3	3.9364E-11	2.140555514
C1orf162	4.08107E-12	2.14044376
CXorf21	3.54616E-11	2.139084841
MS4A14	1.48234E-09	2.139042034
CD6	2.93685E-08	2.139003977
GFPT2	5.38078E-08	2.138646184
CD84	6.03089E-08	2.138192111
TNFSF4	5.39594E-10	2.13799492
GPRIN3	2.6268E-11	2.137954307
ATP8B1	5.47093E-11	2.137805803
KDELR3	1.26615E-06	2.137157327
FAM78B	1.6056E-07	2.136262718
TBXAS1	8.03771E-13	2.134920207

HLA-DQA1	2.78589E-05	2.134419554
C16orf54	4.57803E-13	2.133612564
RUNX1	5.08392E-10	2.132765876
ANO1	2.03129E-06	2.132599754
PLEK	3.04418E-11	2.131860558
CCR4	1.07244E-08	2.130964097
TMEM200B	8.20746E-07	2.130391653
CD300A	5.24857E-11	2.128962602
DAPP1	9.98425E-09	2.128835575
C3AR1	3.37118E-11	2.126962684
PTGDR	9.13422E-10	2.126180387
LTB	2.46634E-08	2.123768628
DMBX1	0.000301267	2.123324368
GCKR	6.18659E-06	2.119860727
MYOF	2.04511E-12	2.119558831
HLA-DRB1	2.39997E-07	2.119135634
ANK1	1.17888E-05	2.116662838
EGR3	1.13027E-06	2.115747041
GBP5	1.51281E-05	2.115713358
RAB38	1.66607E-08	2.115600449
LIMS3	4.37119E-05	2.115128537
CD72	6.59664E-09	2.114378135
RPLP0P2	3.23506E-11	2.11416202
CD109	6.4036E-06	2.113531937
CTLA4	4.62582E-06	2.113106112
HS3ST3A1	0.000398344	2.11044879
C1RL	5.85319E-10	2.109544491
AOX1	0.000134025	2.109316907
GPX8	9.69152E-07	2.107179639
DENND1C	6.80572E-12	2.106940823
SOD3	4.58955E-07	2.106832775
PIK3R6	6.31112E-10	2.105115367
CST6	1.60272E-06	2.10500827
GPR120	2.3699E-10	2.104593224
SLCO2B1	1.19834E-13	2.103642417
DAB2	1.01097E-15	2.103244362
FERMT3	8.02885E-13	2.103011643
CD74	9.3229E-10	2.102481596
TTC39A	4.81476E-07	2.102190014
ZC3H12A	8.2476E-14	2.101014339
DOK3	1.29873E-15	2.099983978
NCF1B	8.19232E-07	2.098804521
ST8SIA4	1.24877E-12	2.098426837
RCN3	1.9444E-10	2.098056311
MXRA8	6.28584E-11	2.096201352
GPRC5A	7.80987E-05	2.09527775
SPON2	1.25178E-06	2.095235765

HGF	6.1893E-05	2.094800118
ZNF831	5.86613E-07	2.094696245
EMILIN1	5.96918E-09	2.094373362
CD36	0.000174461	2.093418917
NXF3	2.85794E-09	2.092746266
PERP	1.24596E-06	2.092701418
FOSL1	2.13551E-06	2.091698048
P2RY6	1.47076E-08	2.091589075
CD28	7.13042E-08	2.091351905
MTL5	6.5082E-08	2.091161308
HS3ST3B1	0.000271763	2.090876535
BAIAP2L1	1.96102E-07	2.090021109
GPR160	7.37454E-11	2.089546624
EDIL3	0.000186858	2.089244621
NLRP2	6.93579E-05	2.088350382
ITGA4	7.5287E-09	2.086959666
NFATC2	1.46783E-05	2.08686818
COL18A1	6.32695E-11	2.086531967
PLBD1	8.38395E-10	2.086513514
TRPV4	4.77049E-08	2.08634204
TMEM176B	2.59104E-06	2.086334165
CD53	4.14431E-10	2.082875263
SPAG4	5.31271E-05	2.082793853
SMAGP	3.48073E-08	2.082355751
GRAP2	1.40229E-08	2.081951278
PVRL4	3.34121E-07	2.081819009
TM7SF4	2.93829E-06	2.081459852
ECM1	2.27621E-13	2.081435083
ITGB3	7.12816E-07	2.0776029
GAPT	5.86588E-08	2.077114383
B3GNT8	1.55367E-09	2.076103069
MYO1D	6.00357E-10	2.075473673
PLEK2	6.85008E-08	2.075185286
C13orf29	2.30336E-08	2.074704121
SERPINB1	5.16829E-12	2.073708345
ODF3B	1.19641E-05	2.072809832
ITGA9	1.33096E-06	2.072778053
ANO4	0.000746373	2.072443856
RIPK3	5.95064E-10	2.071081419
EFEMP1	1.56905E-05	2.071020691
WAS	7.32206E-12	2.070221761
ARPC1B	1.33503E-16	2.068437224
C3orf55	6.94143E-07	2.066640551
EPHX3	1.3117E-06	2.066122841
VEGFC	1.31629E-07	2.065857461
SECTM1	5.25989E-07	2.064400472
PLVAP	7.7448E-08	2.063483146

KLHDC7B	1.65083E-07	2.063106235
NCF2	5.02429E-12	2.062669363
SIRPG	1.95582E-08	2.062144157
CAV1	7.41738E-06	2.060304474
DENND3	8.55382E-14	2.060290989
LEP	2.65288E-09	2.059091565
FMN1	1.89683E-08	2.057105514
ACSL1	8.9394E-14	2.05637394
P2RY10	8.89582E-08	2.056307638
C13orf31	9.02931E-10	2.055787992
SLC2A3	2.48937E-08	2.054651051
WNT4	5.96048E-05	2.053985949
SLC46A2	4.05379E-09	2.051576065
EGR2	4.2183E-07	2.051018627
RAB27A	8.29011E-14	2.049044382
ARSI	0.000614059	2.048155043
GLT8D2	1.90151E-05	2.046514775
CHST4	1.23102E-06	2.04538924
LILRA1	2.08652E-07	2.044377806
POU2F2	2.27344E-09	2.044280986
HLA-DPB1	6.61516E-09	2.042776841
RDH10	1.70919E-07	2.042758894
LOC647121	3.99714E-09	2.042542654
NLRC4	8.11833E-12	2.042435792
RASAL3	4.11564E-10	2.039973817
TGFBR2	1.27326E-16	2.039728725
LTC4S	5.26235E-08	2.038525461
ARHGAP9	1.46059E-12	2.038456027
GBGT1	5.14644E-15	2.038176887
PARVG	3.1537E-13	2.037974881
PLN	1.78692E-06	2.037652495
TMEM106A	1.20467E-13	2.037393793
SLC39A8	1.12983E-12	2.036861717
PDGFRL	4.15221E-05	2.035803596
SYTL5	0.000284315	2.03531231
ADORA3	2.74667E-08	2.034118841
KIAA1217	5.90425E-09	2.033725001
KCNQ1	3.82677E-17	2.033276158
CD86	4.43805E-09	2.032507356
STEAP3	1.78238E-07	2.03219519
UCN2	3.25339E-05	2.032053887
IKZF1	7.14162E-10	2.031188256
CD27	4.29777E-08	2.030863841
DYSF	1.37245E-09	2.030548549
ELF3	5.45817E-05	2.030286332
HSPG2	8.14699E-09	2.029537438
THEMIS	1.14308E-07	2.028532713

MPEG1	6.46702E-09	2.028361972
C17orf60	1.4694E-07	2.028254591
SPI1	6.64291E-12	2.026660518
HLA-DMB	1.08775E-08	2.026613531
UBASH3A	2.01214E-06	2.026449196
KCNE4	6.12631E-06	2.026394893
PLCG2	2.44297E-13	2.024816519
GPNMB	3.32867E-06	2.024811485
HAVCR2	1.17016E-08	2.024576657
HES2	9.42078E-06	2.023793953
NKG7	8.17708E-07	2.023325988
PYROXD2	0.000205283	2.022103165
TMEM176A	2.151E-05	2.021881218
RNASE6	1.70233E-09	2.021779744
FAM20C	1.02255E-08	2.020627949
ANXA2P2	6.5869E-08	2.020552767
ANXA2	4.28501E-08	2.017078385
MPZL2	6.02439E-08	2.016393265
GZMH	5.09847E-05	2.016249001
TLR7	6.35443E-08	2.015739569
SDC1	4.68592E-06	2.015151631
P2RY2	4.62871E-07	2.014407849
FCRL6	8.41491E-10	2.012911166
RASSF5	1.66317E-15	2.01275554
SLC2A14	1.19981E-06	2.011907261
IL32	1.64495E-06	2.011627055
SPRED3	2.9866E-05	2.011572394
PRSS23	4.65017E-09	2.011234757
SAMSN1	7.65621E-09	2.009865557
DUSP23	9.07945E-10	2.009619269
C12orf59	2.73784E-07	2.00756455
HLA-DMA	8.34941E-10	2.007433121
ADAMTS16	0.000124785	2.007240036
IL1RL1	0.000743821	2.004411191
TYROBP	1.08148E-08	2.00281974
ATP2A3	4.92934E-09	2.002792107
FOXC2	3.84936E-08	2.002697581
ARSJ	8.09454E-05	2.00136143
NLRP3	7.03534E-10	2.000482814
LOC254559	0.000465537	0.499363702
CDK4	0.0004831	0.491663424
RCOR2	3.64907E-05	0.486290825
MPPED2	1.65242E-05	0.474668365
C1orf61	1.65157E-05	0.468139515
SALL3	7.7375E-06	0.467349293
MYCN	3.46055E-06	0.466951537
SELV	1.70109E-05	0.465792912

OLIG2	4.48679E-05	0.461789456
ATP13A5	0.000496419	0.451854627
ZDHHC22	0.000293104	0.449456463
CSPG5	5.06429E-06	0.445566146
SOX8	8.82642E-06	0.440558818
CHST9	0.000156228	0.431846393
MSTN	0.000680954	0.420064283
NEU4	4.5447E-05	0.411191055
HES6	2.32518E-08	0.411190296
NKAIN4	9.70713E-05	0.397059538
EMILIN3	5.72416E-05	0.389385438
BCAN	1.44106E-06	0.384246946
BTBD17	5.68533E-06	0.352061067
ASCL1	1.66401E-07	0.344721676
HES5	7.9183E-08	0.266296186
