# Supplementary material for: Silence of Hippo Pathway Associates with Pro-Tumoral Immunosuppression: Potential Therapeutic Target of Glioblastomas
Source: Cells. 2020 Jul 23;9(8):1761. doi: 10.3390/cells9081761 (PMC7464204; doi:10.3390/cells9081761)
Supplement: Supplementary file 1 [file cells-09-01761-s001.zip › cells-838967-proofreading supplementary/Table S2.pdf]

Supplementary Table S2. Upstream regulators suggested by pathway analysis based on 904 genes differentially expressed between the silence of Hippo and active Hippo subgroups of glioblastoma in The Cancer Genome Atlas.

Upstream Regulator	Exp Fold Change	Molecule Type	Predicted Activation State	Activation z-score	p-value of overlap*
lipopolysaccharide		chemical drug	Activated	11.247	7.28E-133
TNF	2.143	cytokine	Activated	9.961	3.49E-129
TGFB1		growth factor	Activated	8.094	8.02E-96
IFNG		cytokine	Activated	7.688	2.75E-95
IL1B	3.061	cytokine	Activated	9.533	5.20E-92
dexamethasone		chemical drug		1.325	3.20E-84
IL4		cytokine	Activated	4.929	1.57E-74
IL10	3.548	cytokine		0.806	2.89E-71
IL6	5.420	cytokine	Activated	7.215	2.87E-70
phorbol myristate acetate		chemical drug	Activated	10.254	3.56E-69
tretinoin		chemical - endogenous mammalian	Activated	8.641	8.16E-62
CSF2		cytokine	Activated	7.860	4.07E-61
NFkB (complex)		complex	Activated	8.844	8.64E-60
STAT3		transcription regulator	Activated	4.583	8.42E-59
IL13		cytokine	Activated	3.818	6.21E-58
prostaglandin E2		chemical - endogenous mammalian	Activated	3.563	1.73E-57
fluticasone		chemical drug		0.694	3.88E-57
Immunoglobulin		complex		-0.947	1.24E-55
IL1A	2.488	cytokine	Activated	7.735	6.50E-54
beta-estradiol		chemical - endogenous mammalian	Activated	2.245	4.51E-51

*, The p-value of overlap was used to rank the significance associated for each upstream regulator. The P-value indicates the significant of the overlap between the genes targeted by the upstrem regulator in the IPKB database and the experimental dataset.
